# Supplementary material for: Offspring production from cryopreserved primordial germ cells in Drosophila
Source: Commun Biol. 2021 Oct 7;4:1159. doi: 10.1038/s42003-021-02692-z (PMC8497528; doi:10.1038/s42003-021-02692-z)
Supplement: Supplementary file 2 — Supplementary information [file 42003_2021_2692_MOESM2_ESM.pdf]

Supplementary information

Offspring production from cryopreserved primordial germ cells in *Drosophila*

Miho Asaoka[1¶], Yurina Sakamaki[2¶], Tatsuya Fukumoto[3 ¶], Kaori Nishimura[4],

Masatoshi Tomaru[4], Toshiyuki Takano-Shimizu[4], Daisuke Tanaka[3*],

and Satoru Kobayashi[1,2*]

[1] Life Science Center for Survival Dynamics, Tsukuba Advanced Research Alliance, University of Tsukuba, Tsukuba, Ibaraki, 305-8577, Japan;

[2] Graduate School of Life and Environmental Sciences, University of Tsukuba, Tsukuba, Ibaraki, 305-8577, Japan;

[3] Research Center of Genetic Resources, National Agriculture and Food Research Organization (NARO), Tsukuba, Ibaraki, 305-8602, Japan;

[4] Advanced Insect Research Promotion Center, Kyoto Institute of Technology, Kyoto, 616-8354, Japan.

*** Corresponding Authors**

E-mail: skob@tara.tsukuba.ac.jp (SK)
masaoka@tara.tsukuba.ac.jp (MA)
fruitfly@kit.ac.jp (TTS)
dtanaka@affrc.go.jp (DT)

¶ These authors contributed equally.

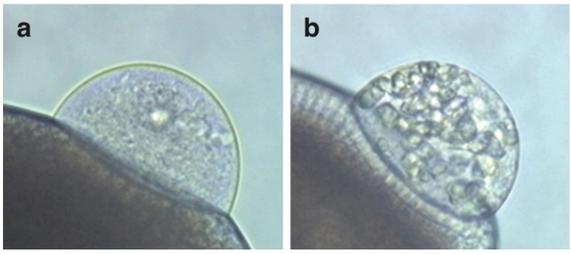

Supplementary Fig. 1: Morphology of PGCs after freeze-thawing. a and b PGCs were freeze-thawed with (**b**) or without CPA (20% EG, 1 M Sucrose) (**a**), and collected in a drop of CPA on the surface of the donor embryo. **a** All PGCs were ruptured and indiscernible (PGC grade 1). **b** Approximately 90% of PGCs remained discernible (PGC grade 3). Please refer to Supplementary Table 1 for grading of PGC morphology.

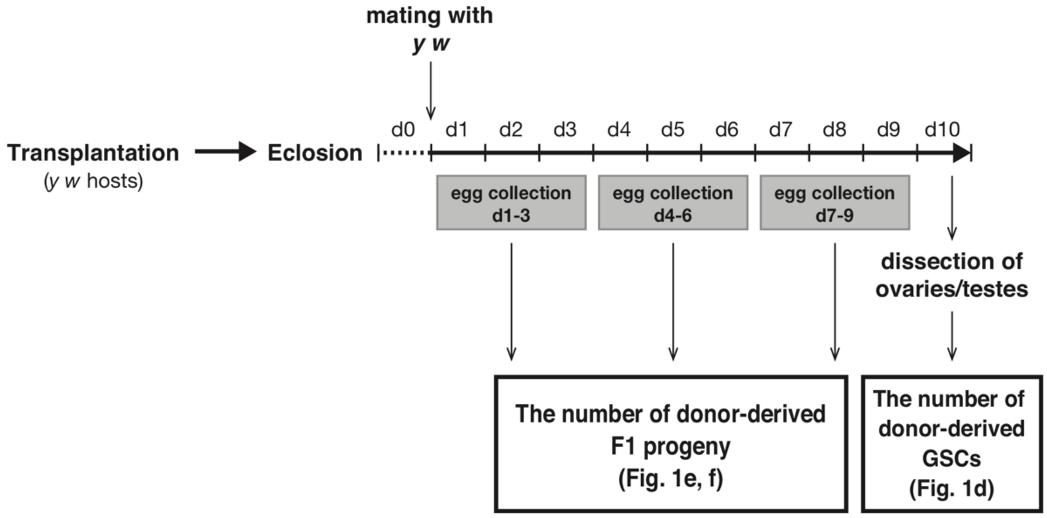

Supplementary Fig. 2: Schematic diagram of experiments for scoring the number of donor-derived F1 progeny and GSCs. F-PGCs, CPA-PGCs, or Naive-PGCs obtained from *EGFP-vas* donor embryos were transplanted into *y w* host embryos, and allowed to develop to adulthood. After eclosion, each female was mated with five *y w* males while each male was mated with four *y w* females. Eggs laid on days 1–3 (d1–3), days 4–6 (d4–6), and days 7–9 (d7–9) were developed to adulthood (F1 progeny). We determined the percentage of adult female hosts producing F1 progeny derived from donor PGCs (red-eye) (Fig. 1e). We also counted the number of red-eyed F1 progeny (Fig. 1f). On day 10, ovaries/testes were dissected from the adult female and male hosts, and were stained for GFP, Vasa, 1B1, FasIII, and DAPI to examine the number of donor-derived GSCs (Fig. 1d).

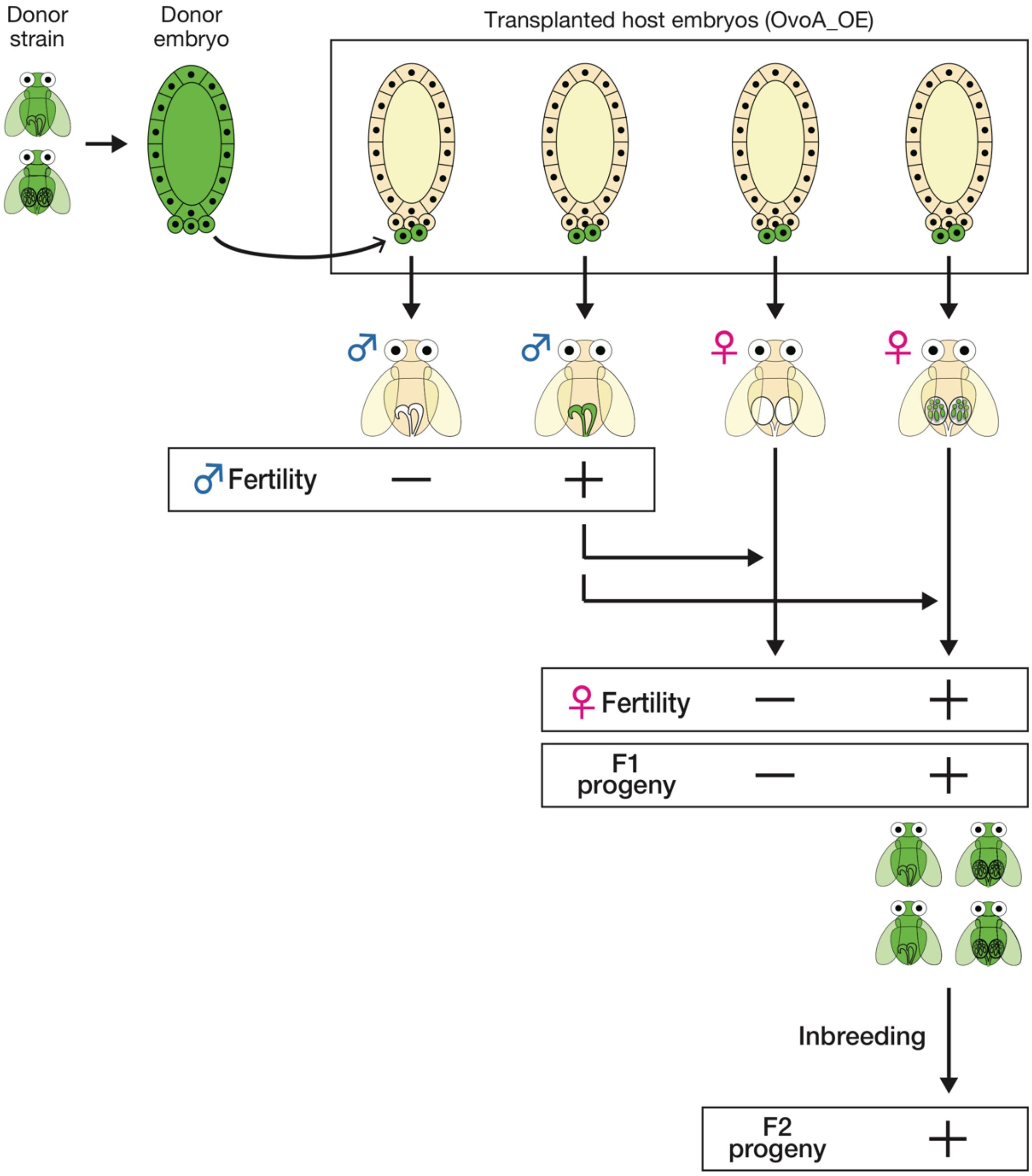

Supplementary Fig. 3: Schematic diagram outlining experiments to revive *M17* strain from cryopreserved PGCs. The agametic OvoA_OE hosts were transplanted with F-PGCs or cryopreserved PGCs that were obtained from *M17* donor embryos. After transplantation, OvoA_OE hosts were allowed to develop to adulthood. Each male host was mated with 4 *y w* females, and incubated in a vial for 4 days. On day 4, the male hosts producing first instar larvae were judged as fertile (Table 2). The single fertile male was mated with single female host, and the female was allowed to lay eggs for 9 days. Female hosts producing F1 progeny were judged as fertile (Table 2). Because OvoA_OE

hosts became sterile, unless donor PGCs were transplanted (Table 2), these fertile adult hosts were producing donor-derived progeny (Fig. 2c). Eggs laid were allowed to develop to adulthood (F1 progeny). F1 females and males were inbred to produce F2 progeny. The phenotypes of the F1 and F2 progeny were examined (Fig. 2d and Table 3). See Progeny Production (Method) for details.

Supplementary Table 1: Effect of cryoprotectants on PGC morphology after freeze-thawing.

Cryoprotectants[a]			PGC grade (n)[c]	
None[b]			1.0	(3)
EG (20%)	+	Sucrose (1.0 M)	3.0	(8)
EG (10%)	+	Sucrose (0.5 M)	2.5	(2)
EG (5%)	+	Sucrose (0.25 M)	1.0	(3)
DMSO (20%)	+	Sucrose (1.0 M)	2.3	(3)
DMSO (10%)	+	Sucrose (0.5 M)	1.7	(3)
DMSO (5%)	+	Sucrose (0.25 M)	1.0	(2)
G (20%)	+	Sucrose (1.0 M)	2.0	(3)
G (10%)	+	Sucrose (0.5 M)	1.0	(3)
G (5%)	+	Sucrose (0.25 M)	1.3	(3)

[a]100 PGCs collected from *EGFP-vas* donor embryos at the blastoderm stage (stage 5) were suspended in each cryoprotectant agent (CPA) and frozen in LN_2 for 20 seconds (sec). After thawing, PGC morphology was observed (Supplementary Fig. 1). EG, ethylene glycol; DMSO, dimethyl sulfoxide; G, glycerol.

[b]PGCs were frozen without using CPA.

[c]After CPA-treatment and freeze-thawing, >50% (PGC grade 3), 5–50% (PGC grade 2), and 0–5% (PGC grade 1) of PGCs remained unruptured and discernible. Mean values of PGC grades obtained from independent experiments are shown. The number of independent experiments (n) is indicated in parentheses.

Supplementary Table 2: Ability of F-PGCs to enter embryonic gonads.

	No. of host embryos[b]			
Donor PGCs[a]	Transplanted	Developing to stage 15	With donor PGCs in gonads (%)	Significance[c]
F-PGCs	40	14	9 (64.3)	
CPA-PGCs	38	18	18 (100.0)	$P_F < 0.05$
Naive-PGCs	42	21	21 (100.0)	$P_F < 0.05, P_{CPA} > 0.5$

[a]Donor PGCs obtained from *EGFP-vas* embryos were CPA-treated and freeze-thawed (F-PGCs), treated with CPA but not freeze-thawed (CPA-PGCs), or free from CPA-treatment and freeze-thawing (Naive-PGCs).

[b]Donor PGCs were transplanted into *y w* host embryos. The host embryos were subsequently allowed to develop to stage 15. Host embryos carrying GFP-positive donor PGCs within the embryonic gonads were counted. Survival rate until stage 15 was 35.0% (F-PGCs), 47.4% (CPA-PGCs), and 50.0% (Naive-PGCs), with no significant difference between these values ($P > 0.1$, two-sided Fisher's exact test).

[c]Significance was calculated vs. F-PGCs (P_F) or CPA-PGCs (P_{CPA}) by two-sided Fisher's exact test.

Supplementary Table 3: Production of donor-derived F1 progeny by each adult host.

Sex of hosts[a]	Hosts[a]	No. of F1 progeny derived from F-PGCs[b]		
		Total	Male	Female
female	f-1	33	15	18
	f-2	28	14	14
	f-3	17	11	6
	f-4	12	5	7
	f-5	11	4	7
	f-6	5	3	2
male	m-1	149	75	74
	m-2	126	77	49
	m-3	40	21	19
	m-4	10	7	3
	m-5	2	0	2

[a]F-PGCs obtained from *EGFP-vas* donor embryos were transplanted into *y w* host embryos and allowed to develop to adulthood. After eclosion, each female was mated with 5 *y w* males while each male was mated with four *y w* females. Eggs laid for 9 d (on days 1–9 after mating) were allowed to develop to adulthood (F1 progeny), and their sexes were identified. Adult hosts producing donor-derived progeny were examined.

[b]The numbers of male and female F1 progeny with red eyes are shown.

Supplementary Table 4: Continuous production of F1 progeny derived from F-PGCs.

Female hosts[a]	No. of F1 progeny derived from F-PGCs[b]		
	days 1–3	days 4–6	days 7–9
f-1	14	7	12
f-2	6	11	11
f-3	7	5	5
f-4	6	3	3
f-5	2	7	2
f-6	3	2	0

[a]F-PGCs obtained from *EGFP-vas* donor embryos were transplanted into *y w* host embryos, and were allowed to develop to adulthood. After eclosion, each female was mated with five *y w* males. Eggs laid for 9 days (on days 1–9 after mating) were allowed to develop to adults (F1 progeny). Ten-day-old adult female hosts were dissected to observe GSCs in their gonads. Adult females carrying GSCs derived from F-PGCs were selected. The individual identification numbers of female hosts correspond to those in Supplementary Table 3.

[b]The number of donor-derived F1 progeny produced by each female host during days 1–3, 4–6, and 7–9 after mating is shown.

Supplementary Table 5: Learning period for our cryopreservation protocol.

Operators	Research groups	Experience of: injection	Experience of: PGC transplantation	Learning period[a]	Efficiency of transplantation[b]: *y w* host	Efficiency of transplantation[b]: OvoA_OE host
A	UT	–	–	—	8.2%	—
B	UT	–	–	3 months	7.7%	—
C	UT	+	+	1 week	9.1%	24.2%
D	KIT	+	–	1 month	11.4%	13.4%

[a]The length of time from the start of learning until donor-derived offspring are stably obtained is shown. Cryopreservation protocol was originally developed by operator A, and passed to B. The protocol was later passed from B to C, and from B and C to D.

[b]The percentage of hosts producing donor-derived progeny is shown.
