# Supplementary material for: Offspring production from cryopreserved primordial germ cells in Drosophila
Source: Commun Biol. 2021 Oct 7;4:1159. doi: 10.1038/s42003-021-02692-z (PMC8497528; doi:10.1038/s42003-021-02692-z)
Supplement: Supplementary file 3 — Description of Additional Supplementary Files [file 42003_2021_2692_MOESM3_ESM.pdf]

Description of Additional Supplementary Files

File name: Supplementary Data 1.
Description: Source data for Figs. 1b, 1d-f, and 2, Tables 1-3, and Supplementary Tables 1-2.
